# Widespread surface ozone reduction triggered by dust storm disturbance on ozone production and destruction chemistry

**DOI:** 10.1126/sciadv.adr4297

**Published:** 2025-05-07

**Authors:** Yunjiang Zhang, Nan Li, Keqin Tang, Ming Wang, Haiwei Li, Ke Li, Bo Zheng, Qiang Zhang, Meng Gao, Jie Fang, Haoran Zhang, Shijie Cui, Junfeng Wang, Mindong Chen, Hong Liao, Xinlei Ge, Didier Hauglustaine, Yves Balkanski, Philippe Ciais, Guibin Jiang

**Affiliations:** ^1^ Collaborative Innovation Center of Atmospheric Environment and Equipment Technology, Jiangsu Key Laboratory of Atmospheric Environment Monitoring and Pollution Control, Joint International Research Laboratory of Climate and Environment Change, School of Environmental Science and Engineering, Nanjing University of Information Science and Technology, Nanjing 210044, China.; ^2^ Laboratoire des Sciences du Climat et de l’Environnement, CNRS-CEA-UVSQ, Université Paris-Saclay, Gif-sur-Yvette, France.; ^3^ Institute of Environment and Ecology, Tsinghua Shenzhen International Graduate School, Tsinghua University, Shenzhen 518055, China.; ^4^ Ministry of Education Key Laboratory for Earth System Modeling, Department of Earth System Science, Tsinghua University, Beijing 100084, China.; ^5^ Department of Geography, Hong Kong Baptist University, Hong Kong SAR 999077, China.; ^6^ School of Atmospheric Sciences, Nanjing University, Nanjing 210033, China.; ^7^ School of Environment and Energy Engineering, Anhui Jianzhu University, Hefei 230601, China.; ^8^ State Key Laboratory of Environmental Chemistry and Ecotoxicology, Research Center for Eco-Environmental Sciences, Chinese Academy of Sciences, Beijing 100085, China.

## Abstract

Natural dust storms are associated with changes to atmospheric photochemical processes, including changes in surface ozone, a critical global air pollutant. Here, we quantified the change in surface ozone during dust storms for regions in China by using a synthesis of measurements and modeling approaches. Our results showed that notable reductions of the average ozone concentration (2.0 to 12.2 parts per billion by volume) were observed during the 12 dust storm events from 2016 to 2023, relative to predust storm levels. The chemical interactions of dust particles with ozone production processes played crucial roles in explaining approximately 13 to 35% of the observed ozone reduction, alongside the impact of intense meteorological disturbances on transport and formation of ozone. Among these interactions, the uptake of ozone, reactive nitrogen, and hydroperoxyl radical by dust particles could substantially contribute to the ozone suppression. This study highlighted the importance of interactions between severe dust pollution and atmospheric photochemistry.

## Teaser

Natural dust storms substantially reduce surface ozone by altering photochemical processes and enhancing ozone destruction.

## INTRODUCTION

Tropospheric ozone is a main air pollutant that substantially impacts human health, ecosystems, and climate. It is produced via the photochemical oxidation processes of gas-phase nitrogen oxides (NO*_x_* = NO + NO_2_) and volatile organic compounds (VOCs) (*1*–*4*). Ozone photochemical regimes are usually classified as VOC limited and NO*_x_* limited based on differences in free radical termination reactions (*5*). Recent studies have introduced a third regime, termed aerosol inhibited. This regime is characterized by the predominant loss of hydroperoxy radicals (HO_2_) through aerosol heterogeneous chemistry (*6*–*9*). In addition, the heterogeneous uptake of ozone precursors and related species by aerosols can also influence ozone production (*1*, *10*, *11*). These impacts are largely dependent on variations in aerosol types and concentrations in the air. Hence, a systematic investigation into aerosol pollution variability and its effects on ozone is crucial for comprehensively understanding the ozone production-destruction processes.

In Earth, approximately one-third of the global land surface is threatened by desertification (*12*–*15*). The exacerbation of desertification intensifies the frequency of dust storms, increasing the particulate matter content in the atmosphere and impacting near-surface ozone (*16*, *17*), particularly in arid and semi-arid regions. Approximately 2 billion metric tons of dust are emitted into the atmosphere annually, with Asia contributing approximately 30% of those emissions (*14*, *15*, *18*). Northwestern and northern China, typifying ozone air pollution in Eastern Asia, frequently experience dusty weather associated with these desert dust emissions (*19*–*23*). Over the past decades, climate change (*13*, *21*, *24*) and anthropogenic activities (*25*, *26*) have been the main driving factors behind expanding desertified areas in Eastern Asia. Some field observations have documented a notable decrease in ozone concentration during dust pollution plumes (*17*, *27*, *28*). Moreover, laboratory simulation experiments have unveiled that some radicals (*29*–*32*) and reactive species (*33*–*37*) can undergo heterogeneous uptake via dust particles (*38*). Chemical model simulations proposed that heterogeneous reactions with dust particles affect ozone in dust source regions, influencing atmospheric photochemical oxidation cycles and reducing ozone concentration (*39*, *40*). However, quantitative evidence elucidating the mechanism by which dust particles in the actual atmosphere reduce ozone concentrations is still lacking.

In this study, we conducted a quantitative analysis of the change in surface ozone across regions in China during dust storms. First, we used machine learning to investigate the effects of meteorological variations and dust particles on the reduction and suppression of ozone during the 12 typical dust storm events from 2016 to 2023. Subsequently, a chemical transport model was used to assess the specific contributions of direct ozone uptake and the indirect effects resulting from the uptake of various radicals and precursors. Last, we evaluated the influence of dust particles on the ozone production sensitivity during dust storm weather conditions.

## RESULTS

### Observed ozone reduction associated with dust storms

**Fig. 1.**
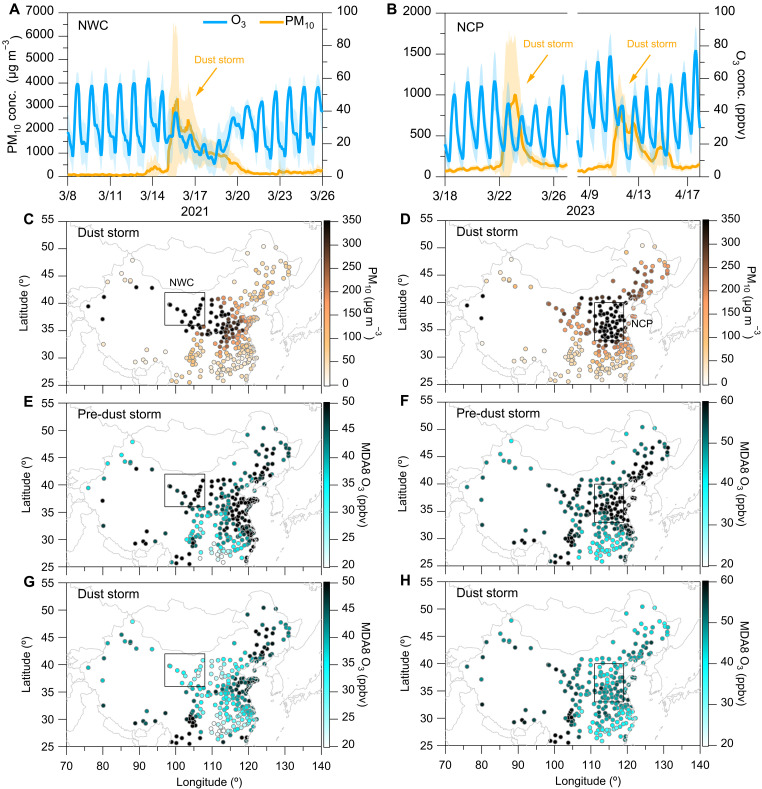
Spatial-temporal patterns of surface ozone during dusty weather conditions. (**A** and **B**) Time series of hourly surface PM_10_ and O_3_ concentrations within the NWC and NCP regions during March 2021 and March–April 2023, delineated by the black box in (**C**) and (**D**), respectively. Shaded areas represent the SD among the selected cities. (**C**) to (**H**) Surface concentrations of PM_10_ and the MDA8 O_3_ during the predust and dust storms periods, respectively. The total 12 typical dust storm events are shown in figs. S1 to S3.

To analyze the spatiotemporal variations of surface ozone under dusty weather conditions, we selected typical dust storm events that occurred in Northwestern China (NWC) and the Northern China Plain (NCP) regions. Because dust emissions are mainly distributed in the coarse particle mode (*41*), the extreme variability in the mass concentration of atmospheric coarse particulate matter (PM_10_) is commonly used as a tracer for dust emission sources (*22*, *42*–*44*). Throughout the dust storm pollution events, PM_10_ concentrations exhibit notable regional increases (*44*, *45*). Using this simplified PM_10_ tracer approach, we identified 12 representative dust storm pollution cases during springtime from 2016 to 2023 (Fig. 1 and figs. S1 to S3). Influenced by dust storms, the PM_10_ concentration in the atmosphere rapidly increased, corresponding to a notable decrease in ozone concentration during the corresponding periods (Fig. 1, A and B, and fig. S1). Compared to conditions preceding dust storms, the average PM_10_ concentration during dust storm episodes in NWC and NCP regions could rise approximately 39 to 1870%, while the maximum daily 8-hour average (MDA8) ozone concentrations decreased by approximately 10 to 44%, respectively (Fig. 1, C to H, and figs. S2 and S3). These results provide direct observational evidence of widespread declines in ozone concentrations during dust storm periods.

From 15 to 19 March 2021, a severe dust storm occurred in the NWC region, representing the most intense dust storm event discussed in this study. Recent researches have attributed this dust pollution event to the influence of a strong Mongolian cyclone, which led to notable emissions of natural dust particles from the dust source region (*22*, *43*). Figure 1A depicts the hourly concentrations of ozone and PM_10_ between the selected cities across the NWC region during the March 2021 dust storm. Under the impact of the dust storm, PM_10_ concentrations surged from 15 March. They persisted for approximately 5 days, coinciding with a decrease in ozone concentration, which reached its lowest level on 18 March. As illustrated in Fig. 1C, the average concentration of PM_10_ during the dust storm period (15 to 19 March) in the NWC region was 1514.3 ± 663.8 μg m^−3^, roughly 20 times higher than the predust storm period (8 to 12 March). This is approximately 30 times higher than the World Health Organization (WHO) air quality guideline of 50 μg m^−3^ (*46*). As shown in fig. S4, a comparison of the diurnal variations in PM_10_ concentrations between the two periods (predust storm versus dust storm) for March 2021 indicates that daily average PM_10_ concentrations during the dust storm were approximately 13 to 21 times and 4 to 7 times higher in the NWC and NCP regions, respectively, relative to predust storm levels. Two extremely intense dust storm events occurred in the NCP region from 22 to 25 March and 11 to 15 April 2023. During these events, daily average PM_10_ concentrations surged to nearly 1000 μg m^−3^ (Fig. 1B), covering almost the entire NCP region (Fig. 1D). These dust storm occurrences could be attributed to Mongolian cyclones transporting dust aerosols from Mongolia and the Taklimakan Desert to northern China (*44*).

Compared to the predust storm period, the average ozone concentration in the NWC region decreased by 11.5 ± 2.8 parts per billion by volume (ppbv) during the March 2021 dust storm (Fig. 1A). The concentration of MDA8 ozone before this dust storm event was 53.0 ± 4.1 ppbv, while it decreased to 30.0 ± 4.8 ppbv during the dust storm event, representing an approximately 43% reduction compared to the predust storm period (Fig. 1, E and G). Similarly, ozone concentrations rapidly decreased during the two dust storm periods in the NCP region (Fig. 1B). Compared to the predust storm period, the average MDA8 ozone concentration in this region decreased by 12.3 ± 3.6 ppbv. Notably, the largest ozone reduction occurred during daytime hours (fig. S4), suggesting that dust particles may affect photochemical processes of ozone production. These observations validated the decrease in ozone concentration and its variation patterns during continuous dust storm events from an observational perspective.

As shown in figs. S5 and S6, the average concentrations of NO_2_ and CO in the NWC region before the March 2021 dust storm were approximately 17.1 ± 5.3 ppbv and 0.7 ± 0.3 parts per million by volume (ppmv), respectively. During the dust storm, the average NO_2_ concentration decreased to 12.5 ± 5.3 ppbv, while change in CO was negligible. A similar pattern was observed during the March to April 2023 dust storm events. In the NCP region, the average concentrations of NO_2_ and CO were around 28.5 ± 8.5 ppbv and 0.6 ± 0.1 ppmv during the predust storms, respectively. During the dust storms, the average NO_2_ concentrations slightly dropped to 27.6 ± 6.0 ppbv, with CO levels remaining stable. Diurnal variations (see fig. S4) revealed a noticeable drop in NO_2_ concentrations during traffic rush hours in the March 2021 event, indicating a potential reduction in anthropogenic emissions, such as vehicular exhaust, during the dust storm. CO, an inert gas at regional scales, showed a limited change, which could be used to assess the impact of photochemical processes by examining the ratio of secondary air pollutants to CO (*47*, *48*), as this ratio could help eliminate or minimize the effects of atmospheric dilution (*49*, *50*). During the March 2021 and March to April 2023 dust storm events, the MDA8 ozone-to-CO ratio decreased by approximately 46 and 15%, respectively, compared to before the dust storm. These reductions suggest that, in addition to meteorological influences, atmospheric chemical processes may play a critical role in ozone loss during dust storms.

### Meteorological impact on ozone reduction during dust storms

**Fig. 2.**
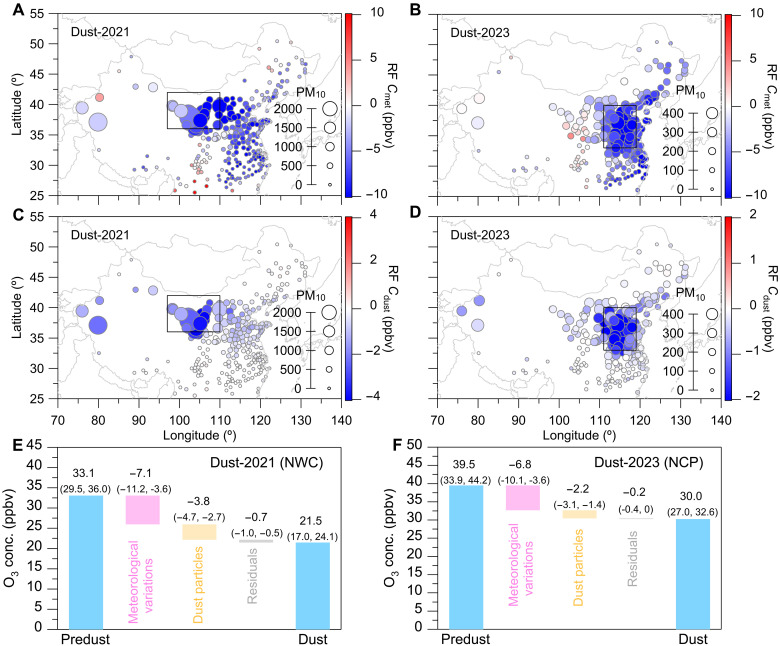
Impact of meteorology and dust particles on surface ozone. (**A** to **D**) Spatial distribution of the average values of $C_{\text{met}}$ and $C_{\text{dust}}$ during the dust storm events (see Materials and Methods). The size of the data points is scaled by the mass concentration of PM_10_ during the corresponding dust storm events. [(A) and (C)] Dust storm in March 2021 (Fig. 1A). [(B) and (D)] Dust storm in March–April 2023 (Fig. 1B). (**E** and **F**) Relative contribution of meteorological variations ($P_{\text{met}}$) and dust particles ($P_{\text{dust}}$) to changes in ozone during the dust storms relative to the predust storms, respectively (see Materials and Methods). Values in parentheses represent the 25th and 75th percentiles for the selected cities in the regions. The 12 typical dust storm events are shown in fig. S7.

To assess the impact of meteorological conditions and dust particles on the reduction in ozone concentration, we applied a machine learning–based approach to estimate the trends in ozone driven by two main factors: meteorological conditions (denoted as $C_{\text{met}}$) and dust particles (denoted as $C_{\text{dust}}$), respectively (see Materials and Methods). The $C_{\text{met}}$ values during the dust storm period primarily represented the influence of the overall meteorological factors (such as atmospheric dispersion and dilution), while $C_{\text{dust}}$ approximated the direct effect of dust particles, including dust-heterogeneous chemical processes. As shown in Fig. 2 (A and B), the $C_{\text{met}}$ during both the March 2021 and March–April 2023 dust storm events exhibit notably negative values over the dust storm–influenced regions. This suggests an important impact of meteorological conditions on ozone reduction under the dust storm conditions.

Figure 2 (E and F) illustrates changes in observed ozone during the dust storms relative to predust storm conditions, along with relative contributions from meteorological variations (denoted as $P_{\text{met}}$) and dust particles (denoted as $P_{\text{dust}}$) (see Materials and Methods). In this analysis, we separated the ozone changes induced by dust photolysis from the broader meteorological effects, isolating this impact specifically to dust particles. Thus, $P_{\text{met}}$ could represent the ozone concentration changes attributed to variations in meteorological conditions, excluding the photolysis effects resulting from dust-radiation impact, while $P_{\text{dust}}$ corresponded to the direct effects of dust particles, including both dust-heterogeneous and dust-photolysis impacts. Similarly, the results for the total 12 typical dust storms are shown in fig. S7. Overall, the $P_{\text{met}}$ values ranged from −9.2 to −1.5 ppbv, explaining approximately 76 ± 7.5% of the total decrease in ozone concentration during dust storms. Particularly evident in the March 2021 (Fig. 2E) and March to April 2023 (Fig. 2F) dust storm events, the $P_{\text{met}}$ was approximately −7.1 ppbv (25th to 75th percentiles: −11.2 to −3.6 ppbv) and −6.8 ppbv (25th to 75th percentiles: −10.1 to −3.6 ppbv), respectively, explaining approximately 69% of the decrease in ozone concentration during dust storm events. These findings emphasize that meteorological disturbances—such as alterations in atmospheric dispersion and dilution—were the dominant factors driving the observed reductions in surface ozone during dust storms.

### Suppression of ozone by dust particles

As shown in Fig. 2 (C and D), the $C_{\text{dust}}$ values exhibited distinct negative trends in the regions affected by dust storms in March 2021 (NWC region) and March to April 2023 (NCP region). Specifically, the magnitude of $C_{\text{dust}}$ was correlated with the PM_10_ concentration during dust storms. As PM_10_ concentration increases, resulting in a larger change in $C_{\text{dust}}$ value, it indicates a more substantial influence of dust particles on reducing ozone concentration during dust storms. As shown in fig. S7, we also calculated the relative contribution of dust particles to ozone decrease in the 12 typical dust storm events. Overall, the $P_{\text{dust}}$ values ranged from −3.8 to −0.5 ppbv, explaining approximately 13 to 35% of the total decrease in ozone concentration during dust storms. Particularly evident in the March 2021 (Fig. 2E) and March–April 2023 (Fig. 2F) dust storm events, the $P_{\text{dust}}$ value was approximately −3.8 ppbv (25th to 75th percentiles: −4.7 to −2.7 ppbv) and −2.2 ppbv (25th to 75th percentiles: −3.1 to −1.4 ppbv), respectively. These reductions explained approximately 22 to 33% of the decrease in ozone during dust storm events. These findings highlight the substantial role of high-concentration dust particles in suppressing ozone concentrations during dust storms.

**Fig. 3.**
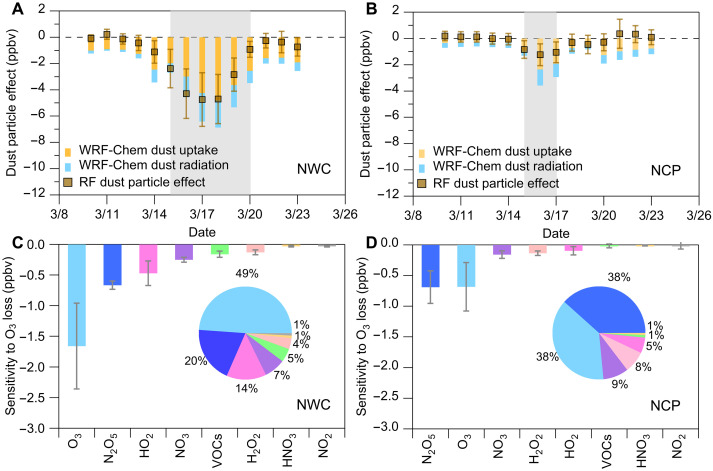
Modeling evidence of surface ozone loss induced by dust particles. (**A** and **B**) Comparison of ozone loss simulated by machine learning and WRF-Chem model during the dust storm (March 2021) for the NWC and NCP regions, respectively. (**C** and **D**) Sensitivity of different chemical pathways to ozone loss was attributed to dust particle uptake simulated by the WRF-Chem model under dust storm weather conditions. The pie chart results refer to the relative contribution of these uptake chemistry pathways. Error bars in (A) to (D) represent the SD among the selected cities in the corresponding regions. The gray shading highlights the period affected by dust pollution.

Solar shortwave radiation is a key meteorological factor influencing atmospheric photolysis reactions, which are critical to ozone photochemistry (*1*, *51*, *52*). Dust particles—by scattering and absorbing radiation—can substantially affect the intensity of shortwave solar radiation in the troposphere (*4*, *18*), thereby reducing photolysis rates in atmospheric photochemical processes (*53*–*55*). As shown in fig. S8, solar shortwave radiation during dust storms was generally lower than during pre-dust storm weather conditions. Specifically, solar shortwave radiation decreased by approximately 13% in the NWC region during the March 2021 dust storm and by approximately 7% in the NCP region during the March–April 2023 dust storm. To further understand the impact of changes in tropospheric shortwave radiation on ozone during dust storms, we also performed a sensitivity analysis turning off radiation impacts of dust in the Weather Research and Forecasting model coupled with Chemistry (WRF-Chem) model (see Fig. 3, A and B, and Materials and Methods). Overall, the total ozone loss simulated by the WRF-Chem model during the March 2021 dust storms over the NWC and NCP regions aligns with the trends predicted by the machine learning model. This comparison between the two methods mutually validates the credibility of the results. Specifically, the ozone concentrations in the NWC and NCP regions decreased by approximately −1.7 and −0.6 ppbv, respectively, due to weakened photolysis during the dust storms. These reductions accounted for approximately 20 and 10% of the observed total ozone reduction during the 2021 March dust storm for the NWC and NCP regions, respectively.

To further explore the dust-induced chemical processes driving this ozone reduction, we focused on the March 2021 dust storm event using the WRF-Chem model to simulate the relative contribution of various heterogeneous uptake pathways to ozone loss (see Materials and Methods). The average total ozone loss due to dust uptake chemistry during the March 2021 dust storms simulated by WRF-Chem model was approximately 3.7 ± 1.1 ppbv in the NWC region and 1.9 ± 1.0 ppbv in the NCP region, respectively. In the NWC region, dust particles exhibit the highest sensitivity through their direct uptake effect on ozone, contributing to approximately −1.7 ± 0.7 ppbv on average. Dust uptake of dinitrogen pentoxide (N_2_O_5_), HO_2_ radical, nitrate radical (NO_3_), VOCs, and hydrogen peroxide (H_2_O_2_) approximately contributed to the ozone variation of −0.7 ± 0.1, −0.5 ± 0.2, −0.3 ± 0.04, −0.2 ± 0.1, and − 0.1 ± 0.04 ppbv, respectively (Fig. 3C). In the NCP region, the dust uptake of N_2_O_5_ emerges as an important chemical pathway for ozone loss during the dust storm, contributing to approximately −0.7 ± 0.3 ppbv (Fig. 3D). Other major contributors via dust uptake pathways include ozone itself (−0.8 ± 0.3 ppbv), NO_3_ (−0.2 ± 0.1 ppbv), H_2_O_2_ (−0.1 ± 0.04 ppbv), and HO_2_ (−0.1 ± 0.1 ppbv), respectively. Notably, N_2_O_5_, an important reactive nitrogen species, uptake by dust particles accounted for an important portion of the total WRF-Chem–simulated ozone loss, contributing approximately 20% in the NWC region and 38% in the NCP region. Similar regional differences in aerosol uptake of N_2_O_5_ have been observed in prior studies on anthropogenic aerosol effects on ozone (*52*). Direct observations of high N_2_O_5_ concentrations in polluted environments have demonstrated its importance in atmospheric chemistry cycles (*52*), particularly in urban regions of eastern China such as Beijing (*56*, *57*), Ji’nan (*58*), and Nanjing (*59*). The heterogeneous chemistry of N_2_O_5_ represents a critical pathway for reactive nitrogen removal (*60*, *61*) and potentially affecting cycling of photochemical processes (*3*, *59*, *62*, *63*). For instance, the loss of N_2_O_5_ through uptake by dust particles during night could reduce the following daytime re-release of NO_2_ (*61*). Our simulation results suggest a pronounced effect of reactive nitrogen uptake by dust particles on ozone production during dust storm events, particularly in the NCP region. In addition, the uptake of HO_2_ by dust particles could be regarded as another radical termination reaction in the ozone formation process (*1*), thereby weakening net ozone production (*6*–*8*). A comparison of ozone reduction caused by dust-induced weakening of photolysis rates with that resulting from dust heterogeneous uptake during the March 2021 event (Fig. 3, A and B) revealed that the simulated ozone loss from heterogeneous uptake was approximately 2 to 3 times greater than that from photolysis weakening.

**Fig. 4.**
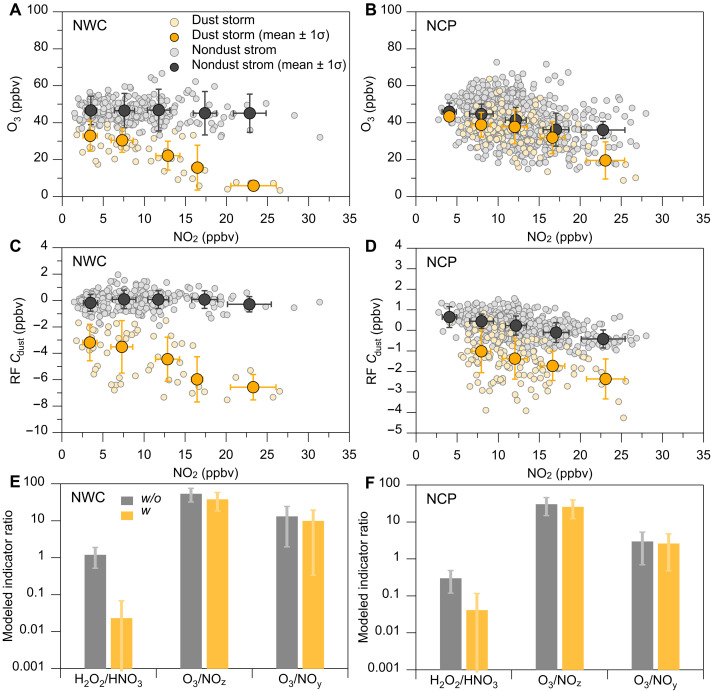
Impact of dust particles on surface ozone production sensitivity. (**A** and **B**) Daytime (1200–1700) relationship between the observed average ozone and NO_2_ during the dust and nondust storm periods throughout the entire March 2021 period over the NWC and NCP regions, respectively. Solid orange dots represent data during the dust storm period. (**C** and **D**) Daytime relationship between $C_{\text{dust}}$ and NO_2_ concentration during daytime. Hollow gray dots represent all data points for March 2021, excluding the dust storm days, while solid gray dots denote their respective averages. (**E** and **F**) WRF-Chem–simulated indicator ratios (including H_2_O_2_/HNO_3_, O_3_/NO*_z_*, and O_3_/NO*_y_*) for ozone production sensitivity without (w/o) and with (w) dust uptake chemistry, respectively.

As shown in Fig. 4 (A and B), we analyzed the relationship between daytime average ozone and NO_2_ over the NWC and NCP regions during the 2021 March dust storm. The results indicated that the average values show a slight decrease or relatively flat trend in ozone concentration with increasing NO_2_ concentration during non-dust storm periods in the NWC region. Differently, there is a decrease trend in ozone concentration with increasing NO_2_ concentration during the dust storm period in this region, reflecting a potential shifting trend in ozone production sensitivity under these extreme dust storm conditions. However, this trend was not substantially observed in the NCP region, likely due to the relatively low levels of dust particles in that area. Figure 4 (C and D) illustrates the impact of NO_2_ concentration changes on the variation of $C_{\text{dust}}$ values in both the NWC and NCP regions, respectively. It showed the relationship between daytime average $C_{\text{dust}}$ and the NO_2_ concentrations during the dust and non-dust storm periods, respectively. The results indicate that the $C_{\text{dust}}$ values tend to be negligible variation as NO_2_ concentration increases in the conditions unaffected by dust storms. However, there was a noticeable downward trend in $C_{\text{dust}}$ values as NO_2_ concentration increases during the dust storm period. These results suggest that the influence of dust uptake on ozone suppression may be more pronounced in the area with higher NO*_x_* levels during extreme dust storm events. Based on the WRF-Chem simulation results (Fig. 4, E and F), it is evident that the ratios of H_2_O_2_/HNO_3_, O_3_/NO*_z_*, and O_3_/NO*_y_*—where NO*_y_* denotes reactive nitrogen and NO*_z_* represents the difference between NO*_y_* and NO*_x_*—under the conditions of dust particle uptake are all lower than those in scenarios without dust particle uptake. During the dust storm, high concentrations of dust particles could uptake substantial amounts of HO*_x_* radicals [including OH, HO_2_, and organic peroxy radicals (RO_2_)], which could consequently weaken the radical termination reactions of HO_2_ through self-reaction step (*2*, *6*, *8*, *30*, *64*, *65*). Our simulation results may suggest that the heterogeneous uptake processes involving high-concentration dust particles could potentially induce the ozone production toward more sensitive to VOC-limited conditions.

## DISCUSSION

**Fig. 5.**
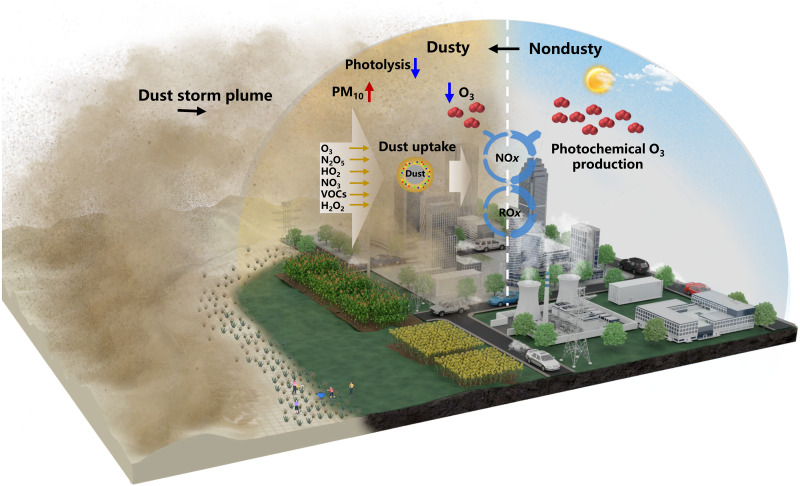
A conceptual scheme for the impact of dust on surface ozone. Dust particles originating from desert areas are emitted and transported into the atmosphere at high concentrations. Because of the scattering and absorption effects of dust particles, solar shortwave radiation is weakened, resulting in a decline in ozone photolysis reactions. The blue cycle represents the standard mechanisms of ozone production via NO*_x_* and RO*_x_* cycles, indicating the sensitivity of ozone production to these cycles with (thin lines, in dust-affected weather) and without (thick lines, in dust-free weather) dust uptake chemistry. The diagram also highlights the reactive species and radicals involved in the chemical loss of ozone through dust uptake processes.

Our findings demonstrated the substantial reduction of surface ozone production coincident with dust storms, insights from the 12 typical dust storm events from 2016 to 2023 in China. This reduction could be primarily attributed to two key factors: meteorological variations and the effect of dust particles. Among these, meteorological disturbances are the dominant contributors to surface ozone reduction during dust storms. The role of dust in modulating surface ozone levels is visualized in Fig. 5, where the occurrence and transport of dust storms directly influence particulate pollution and atmospheric chemical processes. Dust-photolysis interactions, particularly through their suppression of photolysis rates, emerge as one of crucial mechanisms for the observed decline in ozone. In addition, the uptake of ozone, reactive nitrogen, and hydroperoxyl radical by dust particles could substantially contribute to ozone loss. Sensitivity analyses suggest that these uptake processes could influence the complex ozone production-destruction chemistry.

In recent years, ozone pollution in China has become increasingly severe, with both ozone concentrations and the frequency of ozone-polluted days rising steadily. Concurrently, the onset of ozone pollution has occurred earlier, and its duration has been prolonged. In many regions, the first ozone-polluted days now occur as early as March or even February (*66*). This trend highlights the growing importance of investigating the impact of dust on ozone levels under current environmental conditions. Moreover, although ozone concentration in spring may not reach its peak level typically observed in summer, the sustained increase in ozone level during this period can exacerbate the formation of secondary air pollutants, including secondary inorganic and organic aerosols. Hence, examining the influence of dust on ozone is crucial for the future management of complex atmospheric pollution.

Under the context of China, our study could also offer valuable insights into global dust pollution trends. For example, Mongolia and northwest-north China are representative regions that experience frequent dust storms. Recent studies have highlighted a notable decrease in dust activity in this region since the 1970s, mainly attributed to weakened surface winds, increased vegetation cover, and enhanced soil moisture (*21*, *67*, *68*). However, the frequency of dusty weather occurrences in northwest China has risen noticeably in recent years (*19*, *43*, *44*, *69*). One contributing factor is the substantial dust emission from Mongolia’s dust source areas (*24*, *43*). In addition, specific dusty weather conditions, such as the influence of the Mongolian cyclone, have generated strong surface winds, thereby intensifying dust emissions and transport (*24*, *43*). In the past decade, because of factors such as excessive grazing and unregulated mining, the extent of land desertification in Mongolia has remained high, with a deepening severity (*69*). Consequently, these increased dust storm occurrences have substantial implications for recent and potentially future ozone budgets in China. Our study highlighted the complex feedback mechanisms linking dust storm disturbances to surface ozone dynamics. Dust storms have the potential for global environmental impacts through intercontinental transport (*70*, *71*). In the context of ongoing climate change and increasing anthropogenic activities, it could be emphasized the importance of continued research on global dust pollution trends and their effects on atmospheric oxidation capacity and broader atmospheric photochemical cycles (*72*, *73*) as well as their implications for future air quality and climate (*74*, *75*).

## MATERIALS AND METHODS

### Data sources

Hourly surface ozone, PM_10_, CO, and NO_2_ concentration data were sourced from the China National Environmental Monitoring Center and are publicly accessible via the following open-access website (https://air.cnemc.cn:18007/, last accessed on 22 June 2024). The hourly meteorological data, with a spatial resolution of 0.25° × 0.25°, was retrieved from ERA5 reanalysis data provided by the European Centre for Medium-Range Weather Forecasts (ECMWF) and can be downloaded from https://cds.climate.copernicus.eu/ (last accessed on 22 June 2024).

### Machine learning model

The random forest (RF) algorithm is frequently used for the meteorological normalization analysis of atmospheric pollutant concentrations (*76*–*78*). This statistical methodology revolves around constructing a regression model to mitigate the influence of meteorological conditions on pollutant concentration trends. The central objective is to disentangle these trends, distinguishing those influenced by long-term factors (*76*, *79*) or those driven by short-term variations in anthropogenic emissions (*77*, *80*). Following the completion of the regression model, a reconstructed dataset of meteorological feature variables is generated by random sampling from historical meteorological data. In contrast, other feature variables remain original values. Subsequently, this reconstructed dataset is used for predictions using the established model. The process iteratively undergoes multiple repeats of random sampling and prediction. Ultimately, the meteorologically normalized concentration is calculated as the arithmetic mean of these iterations, formally defined as the outcome of meteorological normalization.

In this study, to assess the influence of dust particles on ozone variations, we introduced the PM_10_ feature variable (serving as a proxy for dust particles) into the model, building upon the framework proposed by previous works (*77*, *81*). Alongside PM_10_, the model incorporates additional input variables, including temporal factors (Unix time, day of year, day of week, and hour of day) and meteorological variables (such as air temperature, relative humidity, shortwave solar radiation, sea level pressure, boundary layer height, total precipitation, and zonal, meridional, and vertical wind speeds at various altitudes). Detailed descriptions of these feature variables can be found in table S1. We built individual RF models for each city and each year from 2016 to 2023, focusing on the period from February to May, which includes dust storm events. Using data from shorter timeframes helps detect and elucidate interventions in ozone time series, aligning with similar strategies proposed in previous studies (*77*, *80*–*82*). We used 300 decision trees, with minimum samples required for node splitting and leaf nodes set at 5. We divided 70% of the dataset for model training, while the remaining 30% served as a testing set to assess model performance. We performed fivefold cross-validation and calculated correlation coefficients (*R*) and root mean square error (RMSE) to evaluate the model’s performance and uncertainty. The outcomes of the fivefold cross-validation and testing of the RF models for the average values from 2016 to 2023 were presented in fig. S9, demonstrating the excellent *R* value of 0.9 ± 0.02 and an average RMSE of 6.0 ± 0.5 ppbv between observed and predicted values. Furthermore, comparing the correlation coefficients and RMSE values between the test and cross-validation datasets reveals a relative difference of approximately 0.5 and 2.5%, respectively. These findings could confirm the robust performance of the RF models.

To investigate changes in ozone, three distinct normalization processes were applied using the RF models. First, meteorological normalization was conducted independently, yielding meteorological-normalized ozone concentration results ($C_{\text{demet}}$). The second normalization process simultaneously normalized meteorological conditions and PM_10_ concentrations, resulting in the normalized concentration of meteorology-PM_10_ ($C_{\text{demet}-\text{dust}}$). The third normalization, similar to the first, involved adjusting for meteorological conditions while excluding the normalization of the shortwave solar radiation, producing a meteorologically normalized ozone concentration without normalizing the shortwave solar radiation impact. In each analysis, random sampling and model predictions were executed 1000 times, with the normalized concentration values calculated as the arithmetic mean of these 1000 predictions. To assess the impact of the number of iterations on the evaluation results, we tested different repetitions and examined the distribution changes of the SD of the normalized results obtained (see fig. S10). The results indicated that the SD distribution of the normalized results exhibits characteristics of a normal distribution and gradually converges with an increase in the number of repetitions (*78*). Furthermore, it could be observed that the distribution range essentially stabilizes when the number of repetitions reaches around 1000 iterations. Therefore, setting 1000 as the number of repetitions to calculate the normalized arithmetic mean concentration is reasonable. This number of repetitions is also consistent with the strategies adopted in many previous studies based on machine learning meteorological normalization methods (*77*, *80*, *82*). The RF model and normalization analysis in this study were conducted using the R programming language.

Based on the outputs of the normalization analysis, we calculated the trends in ozone concentrations primarily driven by meteorological conditions ($C_{\text{met}}$) and dust particles ($C_{\text{dust}}$), respectively. The relative change between $C_{\text{demet}}$ and the observed concentration ($C_{\text{obs}}$), was used to calculate $C_{\text{met}}$ (see Eq. 1). Similarly, the relative change between $C_{\text{demet}-\text{dust}}$ and $C_{\text{demet}}$, was used to derive $C_{\text{dust}}$ (see Eq. 2).

$$C_{\text{met}}=C_{\text{obs}}-C_{\text{demet}}$$ (1)

$$C_{\text{dust}}=C_{\text{demet}}-C_{\text{demet}-\text{dust}}$$ (2)

Aerosol-radiation interactions that influence photolysis rates are often considered part of the direct effect of aerosol particles (*51*, *52*, *83*). We further calculated the relative contributions of meteorological variations ($P_{\text{met}}$) and dust particles ($P_{\text{dust}}$) during dust storm periods to the ozone reduction relative to the predust storm levels. This also isolated the radiation effect from the broader meteorological conditions while accounting for the direct dust particle effect. Specifically, the difference between the observed average ozone concentration during the dust storm period [$C_{\text{obs}\left(\text{dur}\right)}$] and the predust storm period [$C_{\text{obs}\left(\text{pre}\right)}$] was regarded as the total change in ozone concentration ($P_{\text{obs}}$) due to the impact of dust storms (see Eq. 3). The difference between the outputs of the third and first normalization steps was considered a proxy for the trend driven by shortwave radiation impacts. The period preceding dust storm events was assumed to be free from dust storm influence and served as the baseline for calculating daytime average values. The daytime differences from this baseline were then used to estimate the dust-mediated photolysis impact ($C_{\text{dpi}}$) associated with the dust storm events. $P_{\text{met}}$ was defined as the difference between the $C_{\text{met}\left(\text{dur}\right)}$ during the dust storm and the $C_{\text{met}\left(\text{pre}\right)}$ before the dust storm, along with $C_{\text{dpi}}$ (see Eq. 4). Similarly, $P_{\text{dust}}$ was determined as the difference between $C_{\text{dust}\left(\text{dur}\right)}$ during the dust storm period and $C_{\text{dust}\left(\text{pre}\right)}$ before the dust storm period, combined with $C_{\text{dpi}}$ (see Eq. 5). Last, the residual values could be calculated using the three variables $P_{\text{obs}}$, $P_{\text{met}}$, and $P_{\text{dust}}$ (see Eq. 6). Results from this method indicate that the average residual among the 12 dust storms was approximately −0.1 ± 0.2 ppbv, accounting for approximately 3 ± 2% of the observed average ozone reduction. The variations in residuals might be attributed to a part of uncertainties inherent in this method

$$P_{\text{obs}}=C_{\text{obs}\left(\text{dur}\right)}-C_{\text{obs}\left(\text{pre}\right)}$$ (3)

$$P_{\text{met}}=C_{\text{met}\left(\text{dur}\right)}-C_{\text{met}\left(\text{pre}\right)}-C_{\text{dpi}}$$ (4)

$$P_{\text{dust}}=C_{\text{dust}\left(\text{dur}\right)}-C_{\text{dust}\left(\text{pre}\right)}+C_{\text{dpi}}$$ (5)

$$\text{Residuals}=P_{\text{obs}}-P_{\text{met}}-P_{\text{dust}}$$ (6)

### Chemical transport model and sensitivity simulations

In this study, we used the regional chemical model WRF-Chem v3.9.1 (*84*) to assess the impacts of dust uptake on ozone. The simulation domain covers China (fig. S11) with a horizontal resolution of 36 km by 36 km. The FNL reanalysis datasets were used to establish the initial and boundary conditions for meteorological parameters (*85*). In addition, global chemical model CAM-Chem data were used to define the initial and boundary conditions of chemical constituents (*86*). The calculation of the photolysis process was conducted using the F-TUV schemes (*87*), while the gas-phase and aerosol chemical reactions were simulated using SAPRC-99 (*88*) and Model for Simulating Aerosol Interactions and Chemistry (*89*) mechanisms, respectively. Detailed model settings can be found in table S2 (*86*–*88*, *90*). Furthermore, biogenic and biomass burning emissions were computed using the Model of Emissions of Gases and Aerosols from Nature (*91*) and the Fire Inventory from NCAR (*92*), respectively. Anthropogenic emissions were obtained from the Multi-Resolution Emission Inventory for China (*93*, *94*).

To better assess the dust uptake effects on ozone, we modified the standard WRF-Chem model, considering both direct and indirect uptake processes on ozone_._ The modified model leads to an examination of a comprehensive array of 10 distinct pathways, including ozone, NO_2_, typical VOCs (CH_3_COOH, CH_3_OH, and CH_2_O), and various reactive species and radicals (HNO_3_, NO_3_, N_2_O_5_, HO_2_, and H_2_O_2_). The pseudo–first-order reaction rate coefficient (s^−1^) (*R*_g_) for the loss of a gas-phase species g was calculated in the modified model. Details of the model are summarized in Supplementary Text and table S3. There remains some uncertainty regarding the dust uptake coefficients of various species and radicals in the WRF-Chem model. For example, the analysis of observational data has found that the uptake coefficient for N_2_O_5_ in urban environments in eastern China falls within the range of approximately 0.02 to 0.1 (*56*–*58*, *95*). Laboratory studies have shown a similarly wide range for the uptake coefficient of N_2_O_5_ by dust particles, varying from 0.01 to 0.2 (*57*). To estimate the model result uncertainty, we varied the base uptake coefficients for different species or radicals by increasing or decreasing them by a factor of five or by using the low and high values recommended from the literatures (Supplementary Text and table S3) (*57*, *58*, *96*–*103*). The results of these uncertainty estimates, as shown in fig. S12, are generally acceptable within the expected range.

Using the modified WRF-Chem model, we further conducted the simulations, which focused on the period (10 to 24 March 2021), including the dust storm, setting 10 parallel simulations to quantify the uptake effects of various pathways on ozone (Fig. 3, C and D). To accurately describe the concentration of coarse particulate matter during the dust storms, this set of simulations assimilates observed PM_10_ concentration to constrain the model calculation. Details of the scenario setting can be found in table S4. To evaluate the model’s performance, we compared the simulated concentrations of NO_2_ and O_3_, derived from a simulation that includes dust uptake chemistry, with their respective observed values (fig. S13). The RMSE and normalized mean bias (NMB) for NO_2_ were 5.5 ppbv and 0.05, respectively, while for ozone, the RMSE and NMB were 11.2 ppbv and 0.17, respectively. Overall, these metrics indicate that the WRF-Chem model performs well in simulating NO_2_ and ozone during dust storm events.

To evaluate the impact of dust uptake processes on ozone formation sensitivity, we used the WRF-Chem model to simulate the changes in the ratios of H_2_O_2_/HNO_3_, O_3_/NO*_z_*, and O_3_/NO*_y_* (Fig. 4, E and F), where NO*_z_* refers to the sum of nitrous acid (HONO), HNO_3_, peroxynitric acid (HNO_4_), N_2_O_5_, NO_3_, peroxy acetyl nitrate (PAN), and organic nitrate (RNO_3_), and NO*_y_* refers to the sum of NO*_x_* and NO*_z_*. The simulations were divided into three scenarios via turning on or off the dust-uptake processes and dust-radiation feedback to compare their respective contributions to ozone formation sensitivity (as shown in fig. S14).
