# Supplementary material for: Widespread surface ozone reduction triggered by dust storm disturbance on ozone production and destruction chemistry
Source: Sci Adv. 2025 May 7;11(19):eadr4297. doi: 10.1126/sciadv.adr4297 (PMC12057657; doi:10.1126/sciadv.adr4297)
Supplement: Supplementary file 1 — Supplementary Text Figs. S1 to S14 Tables S1 to S5 [file sciadv.adr4297_sm.pdf]

Science Advances
AAAS

Supplementary Materials for

Widespread surface ozone reduction triggered by dust storm disturbance on ozone production and destruction chemistry

Yunjiang Zhang *et al.*

Corresponding author: Xinlei Ge, caxinra@163.com

Sci. Adv. **11**, eadr4297 (2025)
DOI: 10.1126/sciadv.adr4297

This PDF file includes:

Supplementary Text
Figs. S1 to S14
Tables S1 to S5

Supplementary Text

Text S1. WRF-Chem model configuration

In the WRF-Chem model, the pseudo-first-order reaction rate coefficient (s^{-1}) for the loss of a gas-phase species *g* due to heterogeneous uptake by dust particles is calculated by Eq. (S1).

$$R_g = \sum \frac{\frac{4}{3}\pi r_i \lambda V_i T_v N_i}{1+Kn\left[\chi+\frac{4(1-\gamma)}{3\gamma}\right]} \quad \text{(S1)}$$

where *i* represents 8 bins in MOSAIC mechanics, r_i, V_i and N_i represent particles effective radius of each bin (cm), the ventilation coefficient and number density of particles in size bin (particles cm^{-3}). V_i and N_i can be calculated by Eq. (S2) and Eq. (S3).

$$V_i = 1 + 36.8r_i + 3012{r_i}^2 \quad \text{(S2)}$$

$$N_i = \frac{M_i \rho_a}{(\frac{4}{3}\pi {r_i}^3)\rho_p} \quad \text{(S3)}$$

Where M_i is the mass mixing ratio of dust particles in each bin ($g\ g^{-1}$). ρ_a is atmospheric mass density ($g\ cm^{-3}$), and ρ_p is the mass density of the dust particles ($g\ cm^{-3}$). T_v in Eq. (S1) is the mean thermal velocity of the diffusing vapor ($cm\ s^{-1}$) calculated by absolute temperature (K) and the molecular weights of gas ($g\ mol^{-1}$) (Eq. S4). The effective mean free path of a gas molecule in air (λ) is defined as Eq. (S5). K_b, δ and p are Boltzmann constant, collision diameter of gas in meter, and gas pressure in Pascal, respectively.

$$T_v = \sqrt{\frac{66512T}{\pi m_g}} \quad \text{(S4)}$$

$$\lambda = \frac{K_b T}{\sqrt{2}\pi\delta^2 p} \quad \text{(S5)}$$

K_n is the dimensionless Knudsen number, obtained from the ratio of λ to r_i. The correction factor for anisotropic movement (χ) calculated from K_n according Eq. (S6).

$$\chi = \frac{\frac{4}{3}Kn+0.71}{Kn+1} \quad \text{(S6)}$$

Text S2. Sensitivity of uptake coefficients to simulated ozone production

The selection of the uptake coefficient significantly affects the efficiency of heterogeneous uptake processes. To account for this uncertainty, we adjusted the uptake coefficient by a factor of five, or applied the high- and low-range values recommended in the literature, both decreasing and increasing it relative to the base γ value used in this study. These adjustments remain within the observed range of uptake coefficients (table S3). Consistent with the methodology outlined in the

main text, we evaluated the impact of dust heterogeneous absorption on ozone concentration changes under these different absorption coefficient scenarios. Overall, considering the range or uncertainty in the uptake coefficients for reactive species or radicals on dust particles, some potential uncertainties persist in our simulation results (e.g., as shown in fig. S12).

Text S3. Sensitivity analysis of impact of dust-radiation interactions on ozone production

We designed four scenarios to assess the dust direct radiative effects on ozone during dust storms. As shown in table S5, scenario tpm_ron serves as the baseline, with the aerosol radiation feedback mechanism active and total particulate matter fully simulated. In Scenario tpm_roff, the feedback mechanism is turned off, and the difference in ozone concentrations between tpm_ron and tpm_roff represents the contribution of total aerosol radiation feedback to ozone attenuation. Focusing specifically on dust storm events, we also evaluate the role of coarse aerosol radiation feedback on ozone. In Scenario fpm_ron, the aerosol radiation feedback is activated, but coarse aerosol emissions are excluded. Scenario fpm_roff is identical to fpm_ron, except the feedback mechanism is deactivated. The results of (tpm_ron – tpm_roff) – (fpm_ron – fpm_roff) represents the impact of dust direct radiation feedback on ozone. The other model settings are consistent with those applied throughout the main study, except that the aerosol radiation effect is turned off. Additionally, we also tested the impact of dust-radiation feedback on ozone formation sensitivity. The results show that the dust-radiation effects could amplify the simulated ozone formation sensitivity to the VOC-limited conditions, similar with the effects of dust uptake (fig S14).

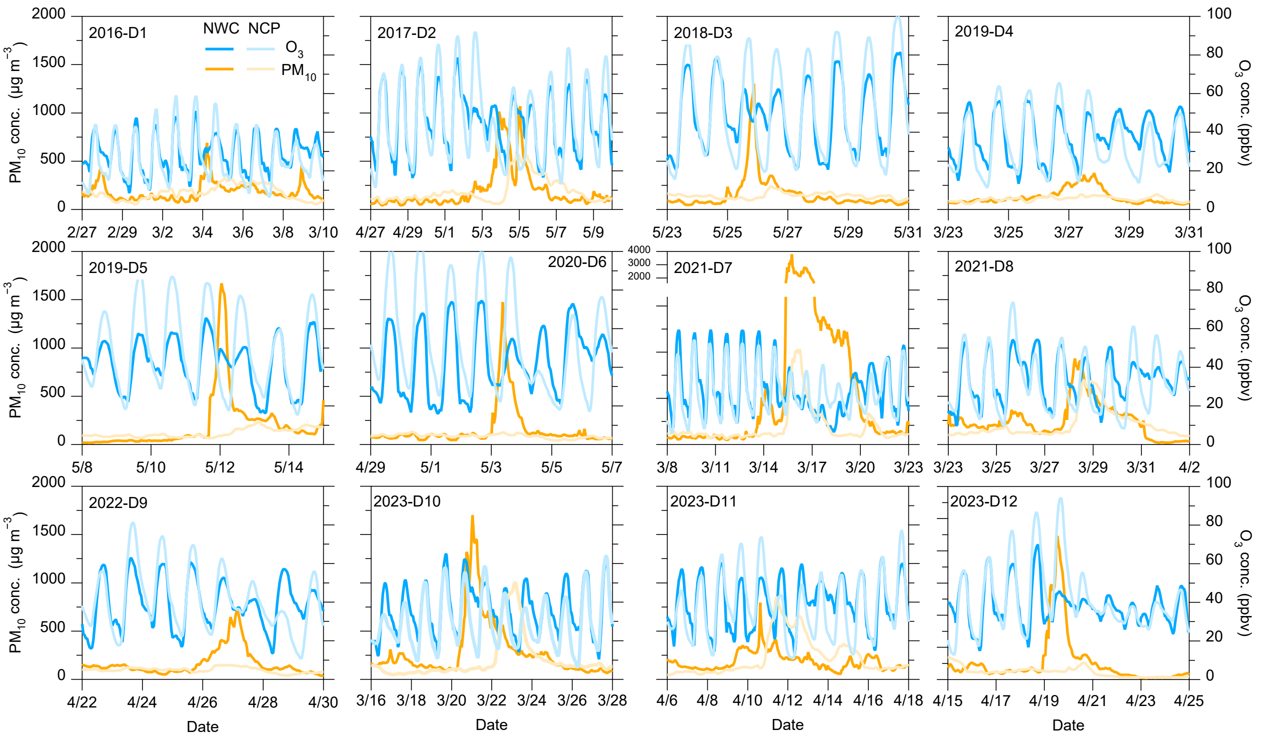

Fig. S1. Temporal variations in aerosol and ozone during dust storm events. Time series of the surface concentrations of hourly PM_{10} and ozone (O_3) for the cities in the Northwestern China (NWC) and Northern China Plain (NCP) regions during the 12 typical dust storm events (D1 – D12), which were selected from the springtime period from 2016 to 2023.

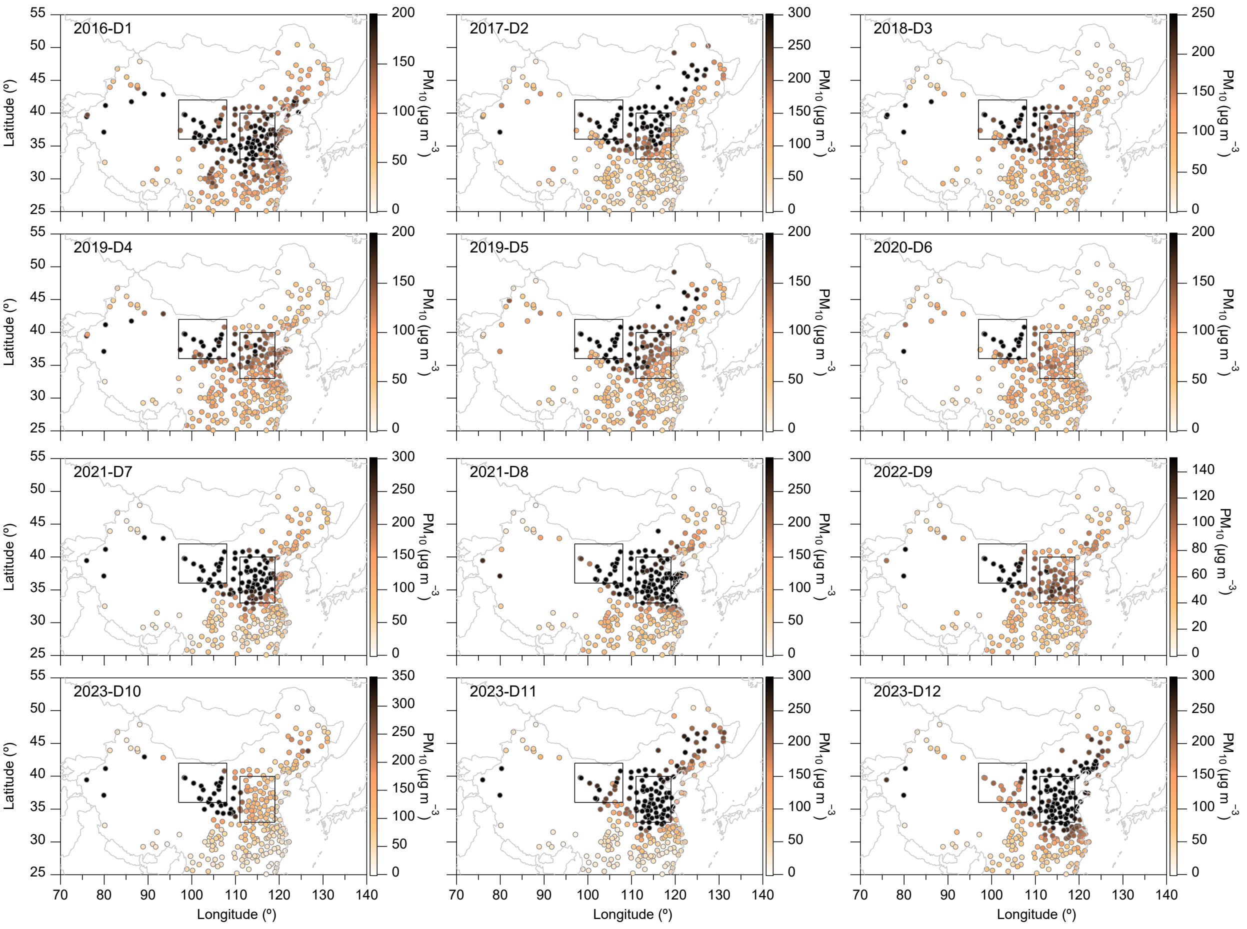

Fig. S2. Spatial distributions of the total 12 typical dust storms. The mass concentration of PM_{10} during the 12 typical dust storm events (D1 – D12) over the NWC and NCP regions during the springtime from 2016 to 2023.

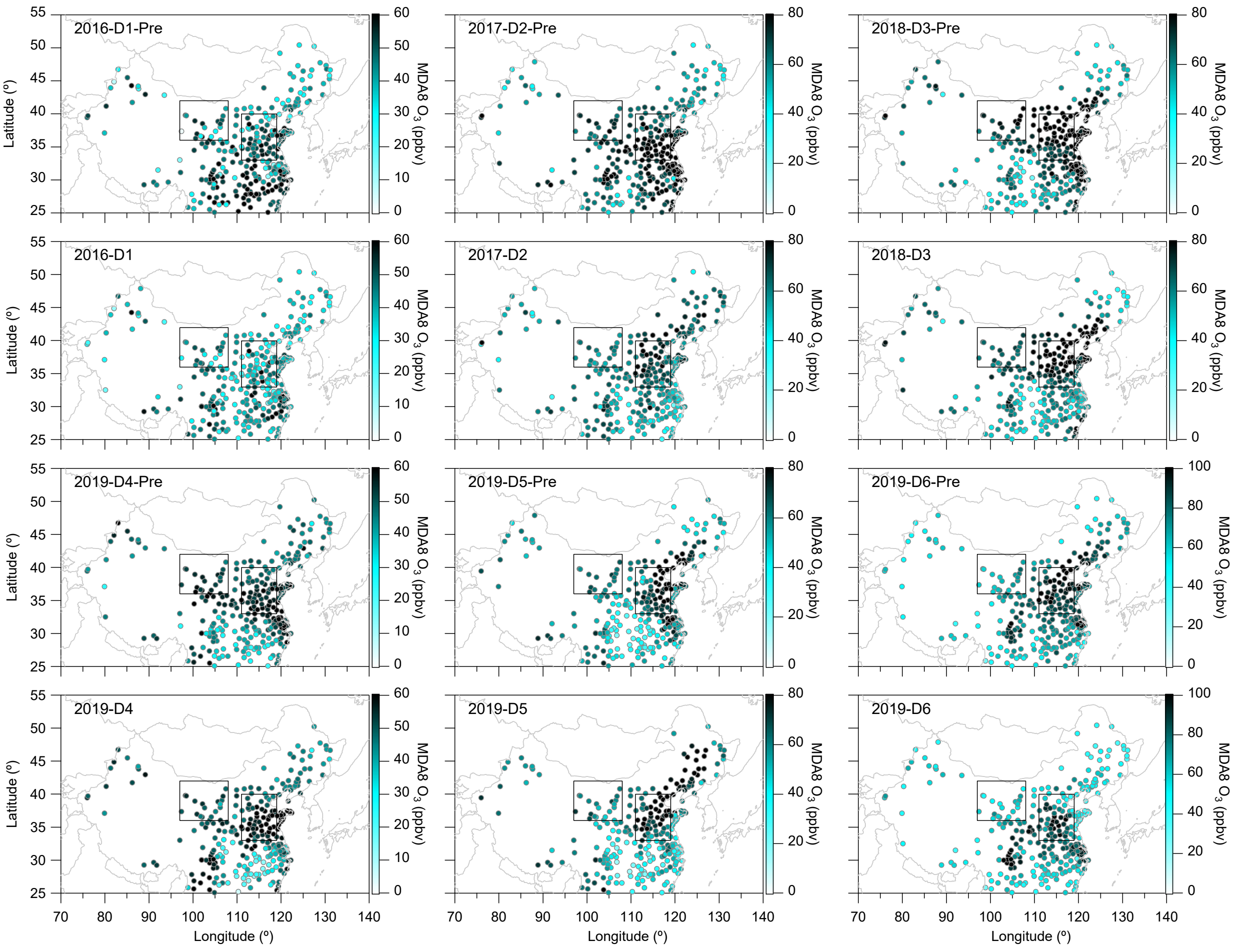

Fig. S3. Observational evidence of surface ozone reduction linked to the dust storms. Changes in surface MDA8 ozone concentrations during the pre-dust and dust storms from 2016 to 2023, respectively.

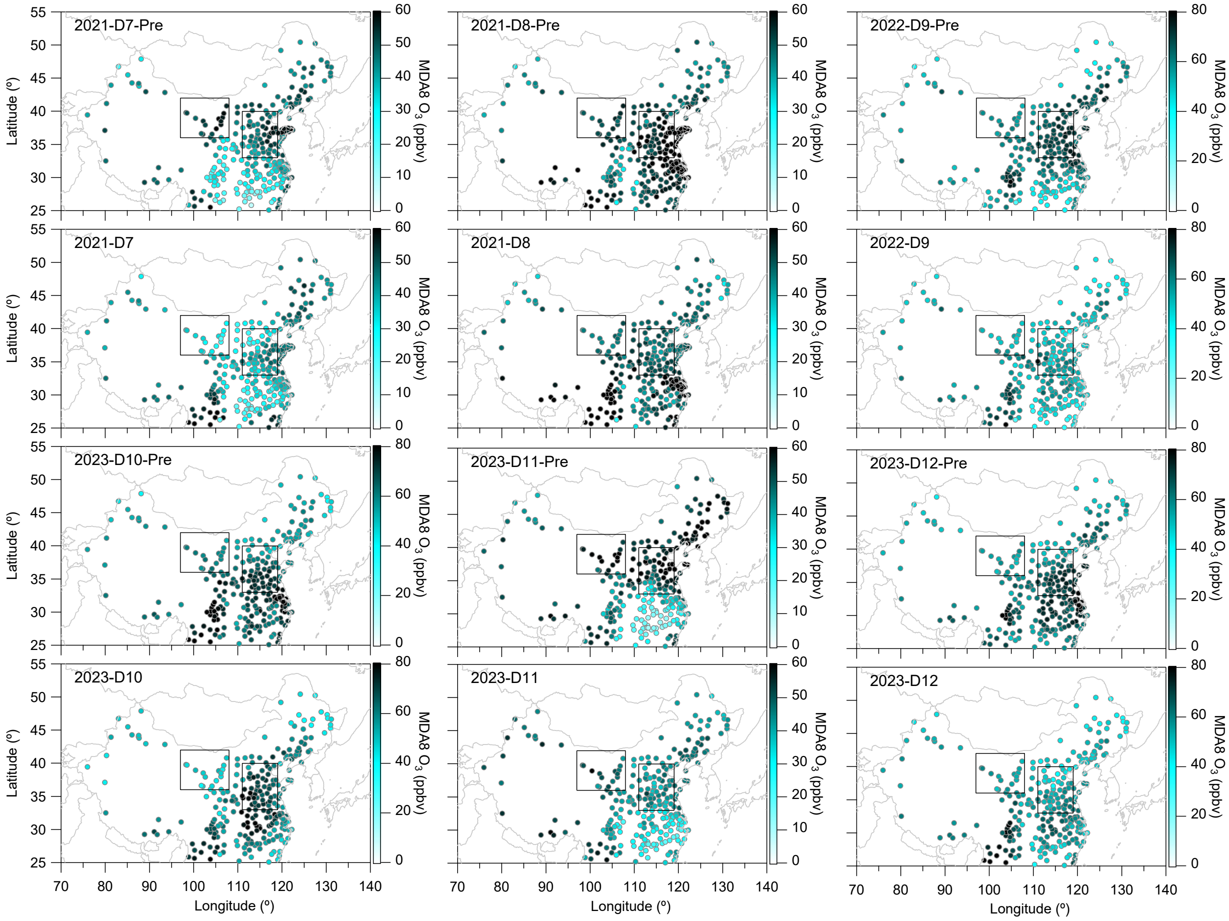

Fig. S3. (Continue).

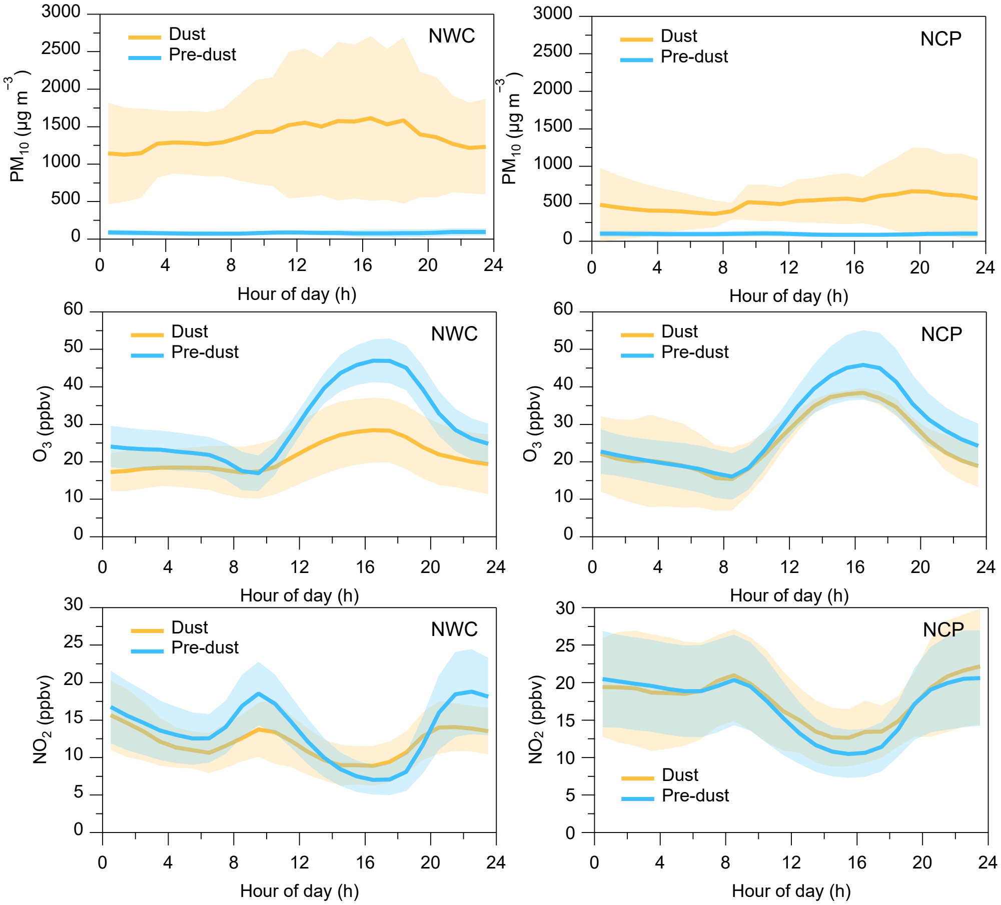

Fig. S4. Diurnal variations of PM_{10}, ozone (O_3) and NO_2. The diurnal variations of observed PM_{10}, O_3 and NO_2 concentrations during the 2021 March (D7) pre-dust and dust storms, respectively. The shaded area represents the standard deviation for the cities in the corresponding region.

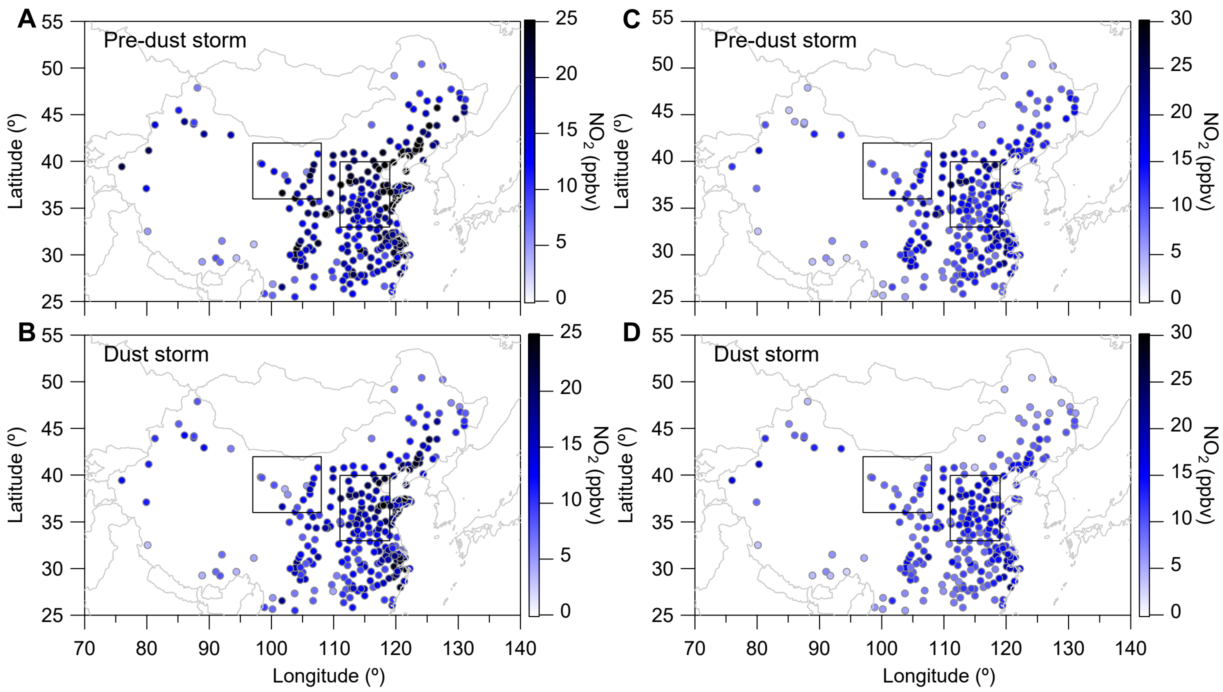

Fig. S5. Comparison of NO_2 between pre-dust storm and dust storm periods. The spatial distribution of NO_2 concentration during the pre-dust and dust storms in 2021 March (D7, **A** and **B**) and in 2023 March-April (D10 and D11, **C** and **D**), respectively.

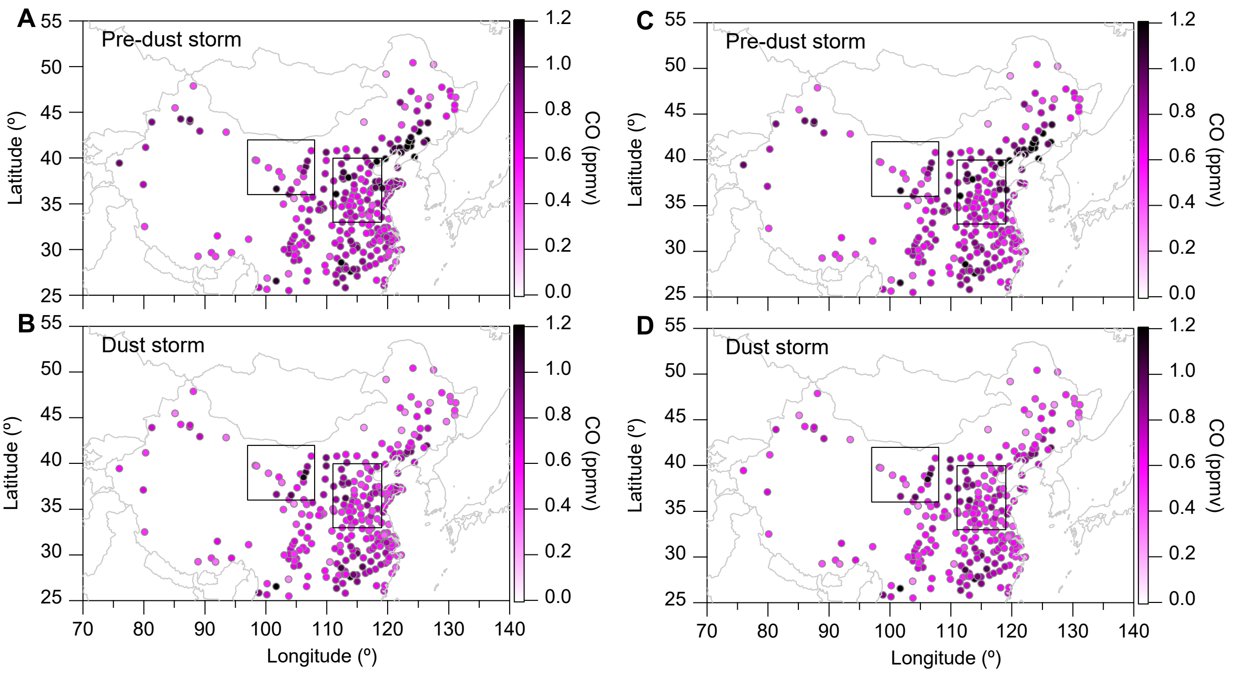

Fig. S6. Comparison of CO between pre-dust storm and dust storm periods. The spatial distribution of CO concentration during the pre-dust storm and dust storm in 2021 March dust storm (D7, **A** and **B**) and in 2023 March-April dust storms (D10 and D11, **C** and **D**), respectively.

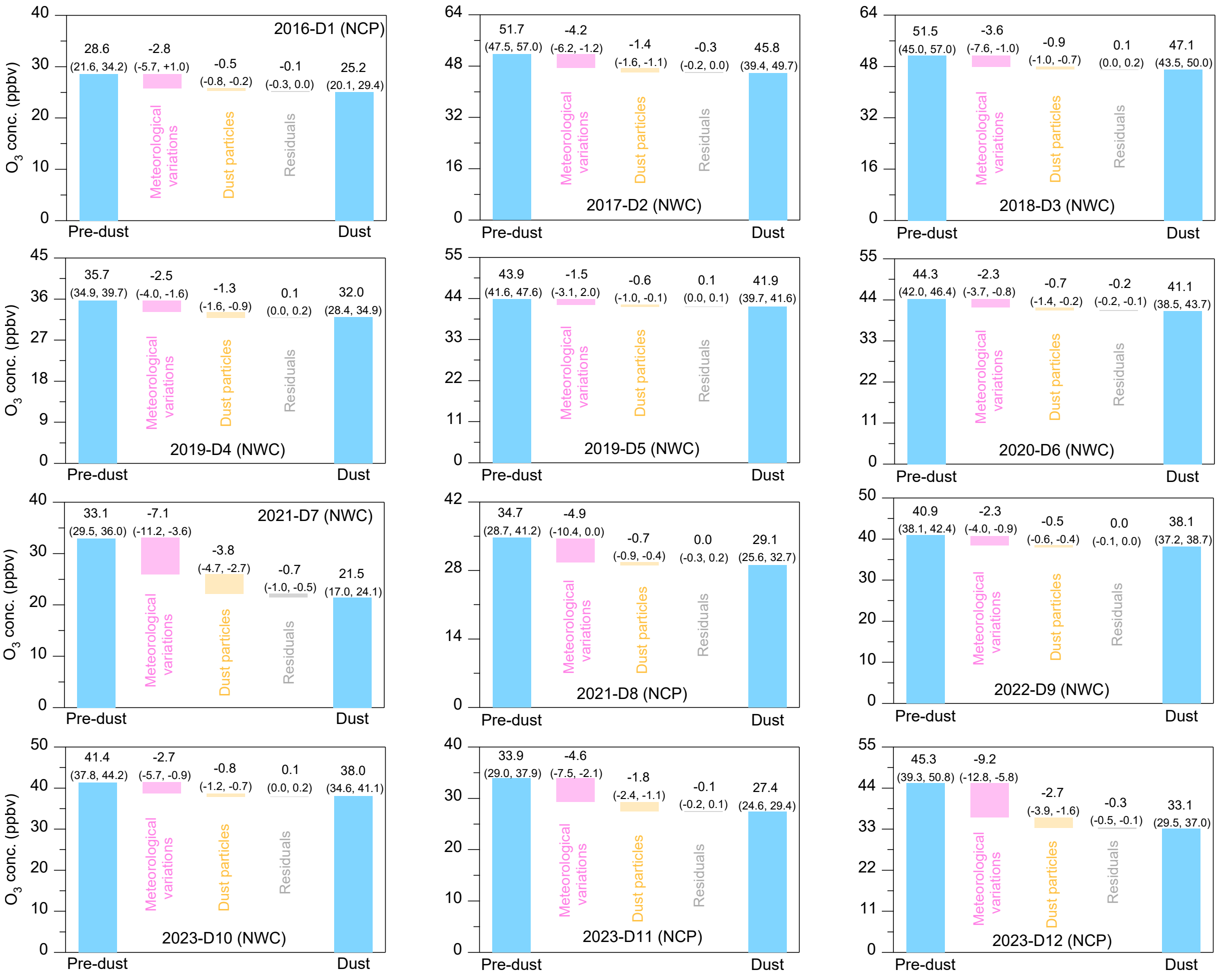

Fig. S7. Drivers of variations in surface ozone during the dust storms. Variations in ozone are driven by meteorological variations ($\boldsymbol{P_{met}}$) and dust particles ($\boldsymbol{P_{dust}}$) during dust storms relative to pre-dust storms and, respectively. Values in parentheses represent the 25th and 75th percentiles for the selected cities in the regions.

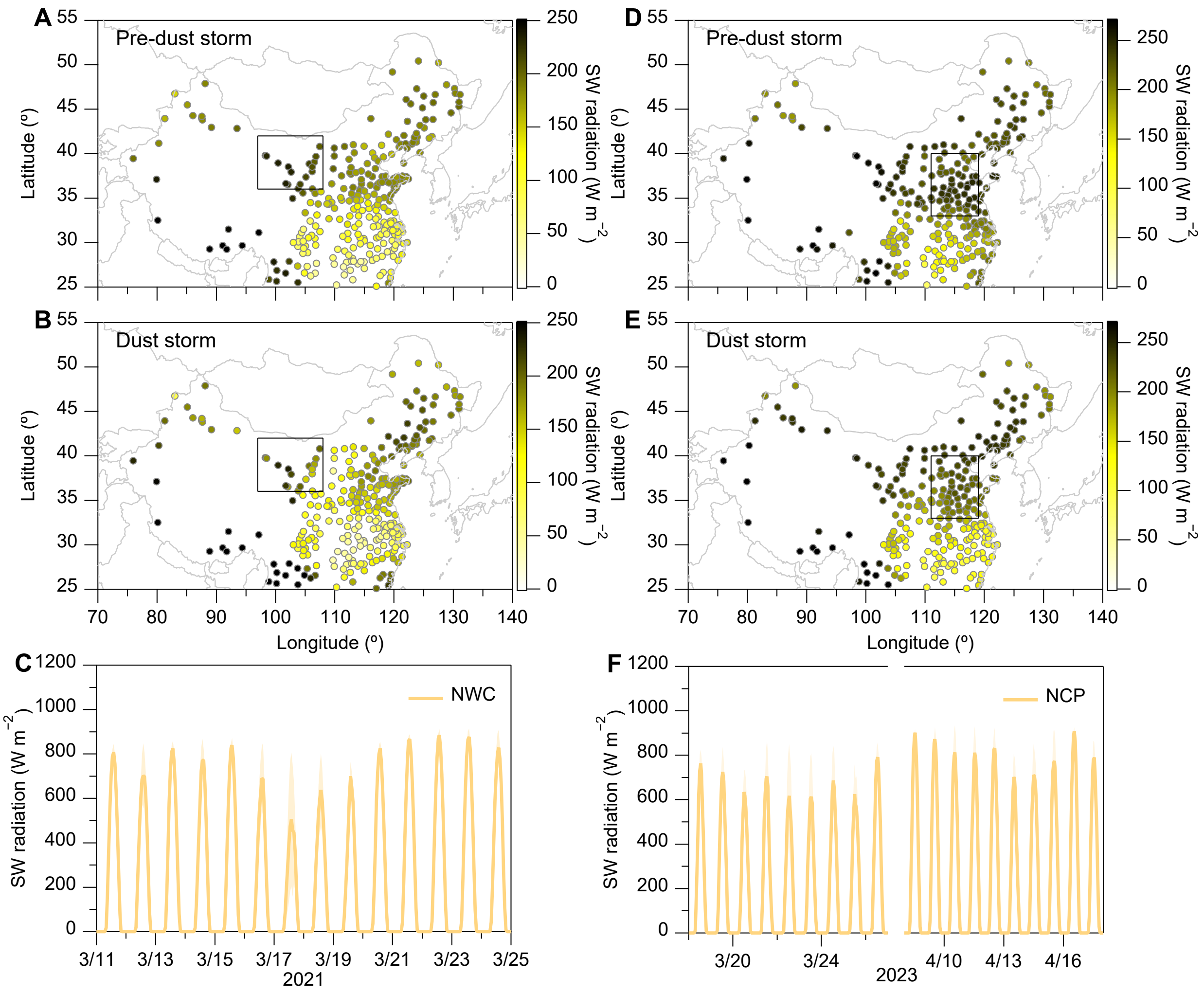

Fig. S8. Spatial and temporal variations of solar shortwave radiation. A and **B**: Spatial distribution of shortwave radiation in the NWC region before (Pre-dust storm) and during the March 2021 dust storm (Dust storm); **D** and **E**: Spatial distribution of shortwave radiation in the NCP region before (Pre-dust storm) and during the March-April 2023 dust storm (Dust storm). **C** and **F**: The corresponding time series characteristics of shortwave radiation variations. Shaded areas represent the standard deviation among the selected cities.

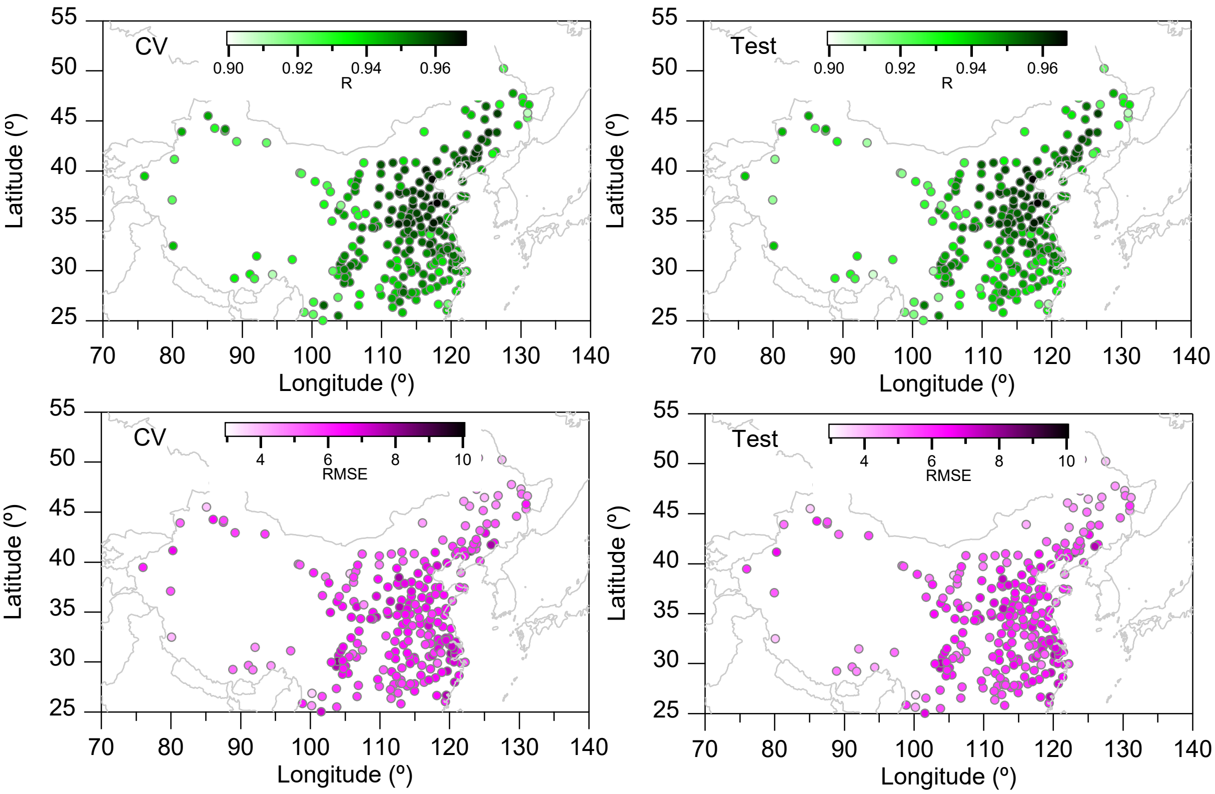

Fig. S9. Evaluation of RF models. The correlation coefficient (*R*) and Root Mean Squared Error (RMSE) between observed and predicted ozone concentrations for the five-fold cross-validation (CV) and the test (Test) sets for the 8-y average values of each year from 2016 to 2023.

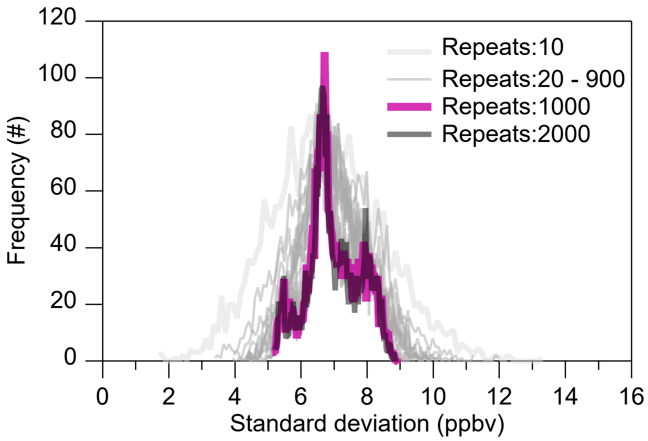

Fig. S10. Sensitivity test on RF model performance. The impact of the number of repetitions in random sampling on the standard deviation distribution of normalized results from the RF model. It illustrates the characteristics of the standard deviation distribution of normalized concentrations of ozone calculated using different numbers of repetitions during the process of predicting random sampling samples with the RF model.

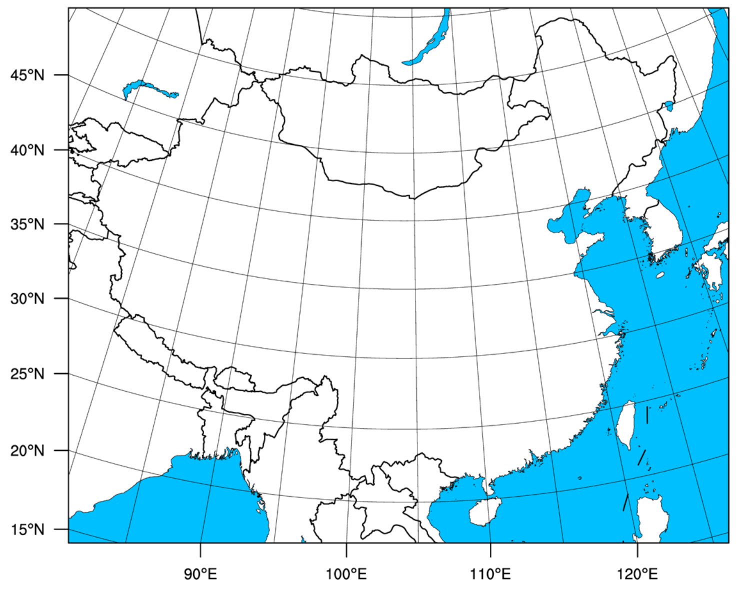

Fig. S11. Simulation domain of the WRF-Chem model. The figure shows the simulation domain of the WRF-Chem model, which has a horizontal resolution of 36 × 36 km².

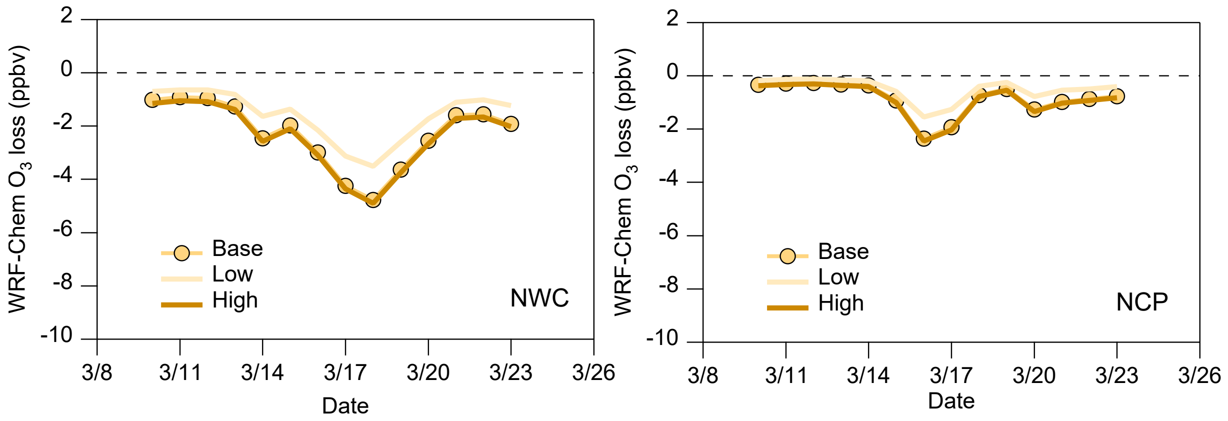

Fig. S12. Sensitivity analysis of dust uptake coefficients. Time series of WRF-Chem-simulated ozone loss due to dust uptake chemistry during the 2021 March dust storm event (D7), where the uptake coefficients were increased or decreased by a factor of 5, or adjusted to the high- and low-range values recommended in the literatures for the NWC and NCP regions, respectively.

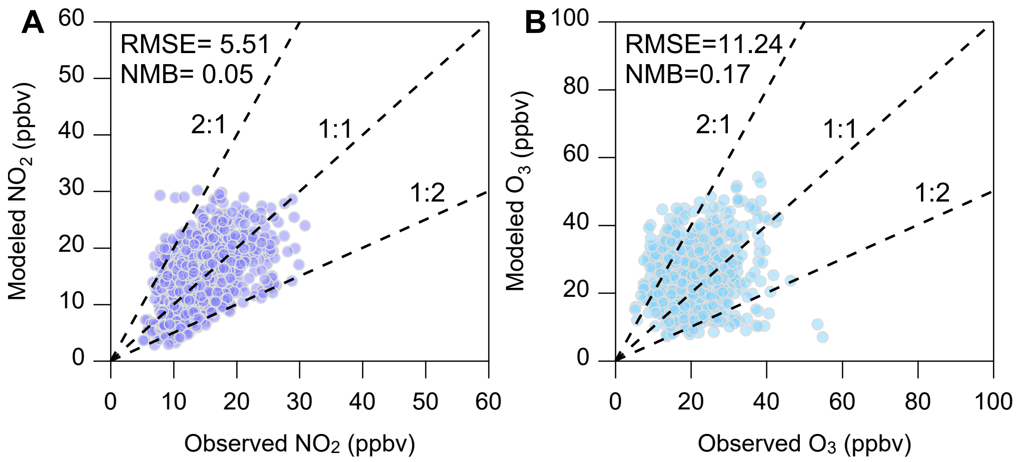

Fig. S13. WRF-Chem model evaluation. Relationships between modeled and observed (**A**) NO_2 and (**B**) O_3, associated with the RMSE and Normalized Mean Biases (NMB) values.

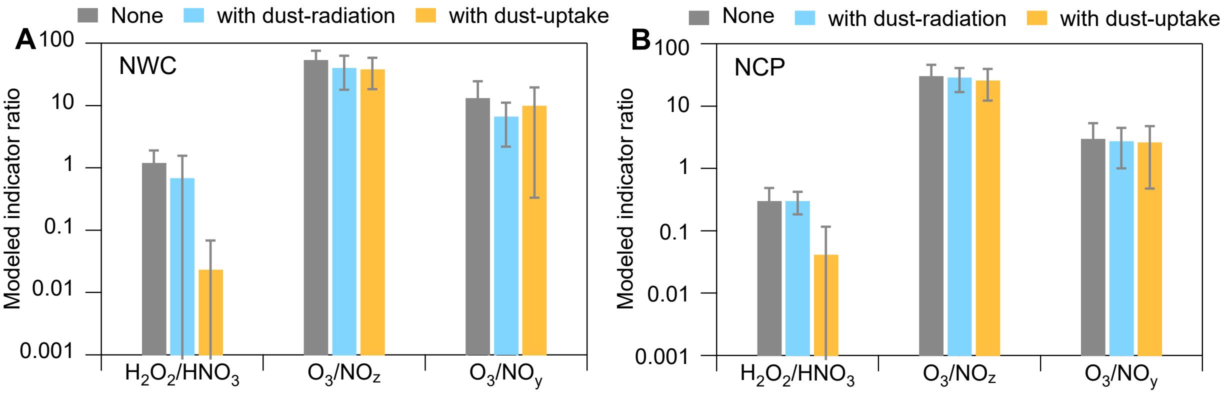

Fig. S14. Analysis of ozone production sensitivity. The WRF-Chem-simulated indicator ratios (including H_2O_2/HNO_3, O_3/NO_z, and O_3/NO_y) are presented for different ozone formation sensitivity: the baseline scenario without dust-radiation feedback and dust uptake effects (referred to as "None"), a scenario with dust-radiation feedback (referred to as "with dust-radiation"), and a scenario with dust uptake chemistry (referred to as "with dust-uptake") for NWC (**A**) and NCP (**B**) regions, respectively.

Table S1. List of predictive features. This table lists the predictive features used in the machine learning model.

Model input variables	Variable description
PM_{10}	Coarse particulate matter ($\mu g\ m^{-3}$)
T2m	Temperature at 2m (K)
SW radiation	Short wave solar radiation ($W\ m^{-2}$)
SP	Sea level pressure (Pa)
RH	Relative humidity (%)
BLH	Boundary layer height (m)
U10	Zonal wind at 10m ($m\ s^{-1}$)
V10	Meridional wind at 10m ($m\ s^{-1}$)
TP	Total precipitation (m)
U850	Zonal wind at 850 hPa ($m\ s^{-1}$)
V850	Meridional wind at 850 hPa ($m\ s^{-1}$)
W850	Vertical velocity at 850 hPa ($Pa\ s^{-1}$)
U500	Zonal wind at 500 hPa ($m\ s^{-1}$)
V500	Meridional wind at 500 hPa ($m\ s^{-1}$)
W500	Vertical velocity at 500 hPa ($Pa\ s^{-1}$)
t_{trend}	Unix time
DOY	Day of year
DOW	Day of week
HOD	Hour of day

Table S2. Model configuration schemes. List of the parameterizations adopted in the WRF-Chem model used in this study.

Options	Parameterization
Microphysics Option	Lin et al. (*90*) scheme
Longwave Radiation Option	RRTMG scheme
Shortwave Radiation Option	RRTMG scheme
Surface Layer Option	MM5 Monin-Obukhov scheme
Photolysis schemes	Madronich F-TUV photolysis
Gas-phase chemical mechanisms	SAPRC99 scheme
Aerosol schemes	MOSAIC scheme

Table S3. Uptake coefficient (γ) of dust for the corresponding reaction pathways. "Base" represents the values used in this study, while "Low" and "High" indicate the low and high ranges recommended from literatures or values manually adjusted by a factor of five, used in the sensitivity analysis to assess the impact of uptake coefficient variations on the modeling results.

**Reaction**	γ			**References**
	Base	Low	High	
O_3+ Dust → P	1×10^{-5}	2×10^{-6}	5×10^{-5}	(*96*)
HNO_3+ Dust → 0.5 NO_x+ P	8×10^{-4}	1.6×10^{-4}	4×10^{-3}	(*97*)
NO_2+ Dust → P	4×10^{-8}	8×10^{-9}	2×10^{-7}	(*98, 99*)
NO_3+ Dust → P	0.23	4.6×10^{-2}	0.23	(*100*)
N_2O_5+ Dust → P	0.05	0.01	0.3	(*57, 58, 100, 101*)
HO_2+ Dust → 0.1 H_2O_2+ P	0.2	0.04	1	(*99, 102*)
H_2O_2+ Dust → P	0.18	3.6×10^{-2}	0.18	(*99, 103*)
CH_3COOH + Dust → P	2×10^{-3}	4×10^{-4}	2×10^{-3}	(*99*)
CH_3OH + Dust → P	1.9×10^{-4}	3.8×10^{-5}	1.9×10^{-4}	(*99*)
CH_2O + Dust → P	3×10^{-5}	6×10^{-6}	1.1×10^{-4}	(*99*)

Table S4. Model chemical uptake pathways. Sensitivity simulation setting for quantifying different impacts of dust uptake pathways. "/" represents not selected, and "√" represents selected.

Scenario	$Path_{base}$	$Path_{o3}$	$Path_{n2o5}$	$Path_{ho2}$	$Path_{no3}$	$Path_{vocs}$	$Path_{h2o2}$	$Path_{hno3}$	$Path_{no2}$
O_3	/	√	/	/	/	/	/	/	/
HNO_3	/	/	/	/	/	/	/	√	/
NO_2	/	/	/	/	/	/	/	/	√
NO_3	/	/	/	/	√	/	/	/	/
N_2O_5	/	/	√	/	/	/	/	/	/
HO_2	/	/	/	√	/	/	/	/	/
H_2O_2	/	/	/	/	/	/	√	/	/
CH_3COOH	/	/	/	/	/	√	/	/	/
CH_3OH	/	/	/	/	/	√	/	/	/
CH_2O	/	/	/	/	/	√	/		/

Table S5. List of configurations for sensitivity experiments. This table lists the configurations for sensitivity experiments designed to quantify aerosol radiation feedback under various scenarios.

Scenario	Aer_ra_feedback	$PM_{2.5}$ emission	PM_{coarse} emission
tpm_ron	On	On	On
tpm_roff	Off	On	On
fpm_ron	On	On	Off
fpm_roff	Off	On	Off
